# Factors associated with the support of pricking (female genital cutting type IV) among Somali immigrants – a cross-sectional study in Sweden

**DOI:** 10.1186/s12978-017-0351-0

**Published:** 2017-08-08

**Authors:** Anna Wahlberg, Sara Johnsdotter, Katarina Ekholm Selling, Carina Källestål, Birgitta Essén

**Affiliations:** 1 Department of Women’s and Children’s Health, International Maternal and Child Health (IMCH), Uppsala University, Akademiska sjukhuset, SE-751 85 Uppsala, Sweden; 2 Faculty of Health and Society, Malmö University, SE-205 06 Malmö, Sweden

**Keywords:** Female genital cutting, Female genital mutilation, Female circumcision, Pricking, Nicking, Diaspora, Migration, Sweden, Somalia

## Abstract

**Background:**

Pricking, classified as female genital cutting (FGC) type IV by the World Health Organization, is an under-researched area gaining momentum among diaspora communities. Our aim was to explore factors associated with being supportive of pricking among Somalis in Sweden.

**Methods:**

In a cross-sectional design, attitudes and knowledge regarding FGC, and measures of socioeconomic status, acculturation, and social capital, were assessed by a 49-item questionnaire in four municipalities in Sweden. Data were collected in 2015 from 648 Somali men and women, ≥ 18 years old, of which 113 supported the continuation of pricking. Logistic regression was used for the analysis.

**Results:**

Those more likely to support the continuation of pricking were older, originally from rural areas, and newly arrived in Sweden. Further, those who reported that they thought pricking was: acceptable, according to their religion (aOR: 10.59, 95% CI: 5.44–20.62); not a violation of children’s rights (aOR: 2.86, 95% CI: 1.46–5.61); and did not cause long-term health complications (aOR: 5.52, 95% CI: 2.25–13.52) had higher odds of supporting pricking. Religion was strongly associated with the support of pricking among both genders. However, for men, children’s rights and the definition of pricking as FGC or not were important aspects in how they viewed pricking, while, for women, health complications and respectability were important.

**Conclusions:**

Values known to be associated with FGC in general are also related to pricking. Hence, there seems to be a change in what types of FGC are supported rather than in their perceived values.

**Electronic supplementary material:**

The online version of this article (doi:10.1186/s12978-017-0351-0) contains supplementary material, which is available to authorized users.

## Plain english summary

The World Health Organization classifies female genital cutting (FGC) into four types. Pricking of the clitoris or labia, classified as type IV, distinguishes itself from types I–III as it involves no removal of tissue. Among African immigrant groups in non-FGC-practising countries, pricking has been reported to have gained support. While the practice of pricking is therefore increasingly discussed in western countries, there is a lack of empirical data on pricking. Although the majority of our respondents opposed all forms of FGC, our study confirms that there is some support for pricking among Somali immigrants in Sweden. The impact of religion, children’s rights, and health were important factors for whether one supported pricking. Motives for supporting pricking differed between Somali men and women. In a globalised world, our study contributes with new knowledge on the values underpinning the practice of pricking, knowledge of importance for informed decision-making among policy-makers and health care practitioners.

## Background

Due to migration, female genital cutting (FGC) has become an emerging maternal and child issue throughout the world. Around 60,000 Somali-born men and women live in Sweden, where all forms of FGC are criminalised, which makes them the largest immigrant group from a country where FGC is traditionally practised [1]. Of girls who have undergone FGC in Somalia, 63% have had their genitals sewn closed, 25% have been cut with flesh removed, and 5% have had pricking with no flesh removed [2]. However, among the Somali diaspora in Sweden (as well as in London and Toronto), the practice of pricking has been reported to have gained support [3, 4]. Pricking of the clitoris or surrounding tissue, in which the skin is pricked with a sharp object and blood may be let, but no tissue is removed and no stitching performed, is classified by the World Health Organization (WHO) as FGC type IV, which is an umbrella term for ‘all other harmful procedures to the female genitalia for non-medical purposes’ [5]. As pricking causes no anatomical changes, it distinguishes itself from FGC types I–III, which include removal of tissue and/or stitching. However, there is a tendency in FGC research and literature not to differentiate between the different types of FGC, or to focus on types I–III [6]. A few studies in Southeast Asia have explored perceptions of and reasons for performing pricking. Within this context, pricking was viewed as a process of socialisation and, for some, as an Islamic practice [7–9]. No physical evidence on the clitoris or labia could be found among women that had undergone pricking [7], and the practice was generally perceived by the communities as non-harmful [7, 9].

Discussions of pricking among policy-makers, international agencies, and researchers are often framed within one of two FGC eradication approaches; harm-reduction or zero tolerance [5]. Depending on the approach, varying conclusions on whether pricking should be allowed are drawn. Within a harm-reduction approach, the use of less extensive forms of FGC, such as pricking, is seen as preferable to other more severe forms of FGC [10]. Allowing pricking to be practised is proposed to act as a first step towards total eradication of FGC [9, 11]. Further, it is argued that a medically safe pricking would neither be physically harmful as it involves no removal of tissue [10, 12, 13], nor a violation of children’s rights [10, 14]. For the above-mentioned reasons, the WHO has been criticised for classifying pricking as a type of FGC [5]. There have been some propositions in Western countries to allow doctors to perform pricking [10, 15]. However, in all cases, the negative reactions against these suggestions from those supporting a zero tolerance approach have been fierce [14–17]. As a result, no changes to allow pricking have been made.

Zero tolerance toward FGC is the prevailing attitude in Western countries. All types of FGC, including pricking, are seen as violations of children’s rights and are thus not allowed [2]. Further, self-reported forms of FGC have been shown to under-estimate the actual anatomical extent of FGC performed [18]. Thus, there is a fear that pricking, described as a replacement for more severe types of FGC, would involve a change in terminology rather than a change in the actual practice of FGC. For this reason, as well as to document changes in practices, the WHO has decided to retain pricking as a type of FGC [5]. However, the zero tolerance approach has been criticised, as it does not recognise the diversity of procedures classified as FGC [9, 10]. Further, parallels between pricking and the usually legal practices of female genital cosmetic surgery and male circumcision are drawn to problematize how pricking is being viewed within a zero tolerance approach [19, 20].

Thus, pricking is a value-laden, yet, under-researched area. Therefore, our aim was to explore important factors for supporting the continuation of pricking among Somalis in Sweden.

## Methods

### Study participants

Somali men and women over 18 years of age were eligible to participate in this cross-sectional survey, performed in the four largest municipalities in Sweden; Stockholm, Gothenburg, Malmo, and Uppsala. Participants were recruited through purposeful sampling at Somali organisations, in public places (such as cafés), at Swedish for Immigrants courses, and in mosques. From participants recruited in these places, snowball sampling was used to reach more participants.

### Data collection

Data on demographics, attitudes and knowledge regarding FGC were collected in 2015 through a validated [21] and pilot tested 49-item questionnaire that had been translated and back-translated from English to Somali (Additional file 1). Questions about FGC were based on its anatomical extent rather than on the WHO classification [18]. Pricking was defined as procedures in which the skin is pricked with a sharp object; blood may be let, but no tissue is removed, and there is no permanent alteration of the external genitalia [5]. The Somali translation of pricking that we used was [Dhiijin aan cad la jarin]. Six Somali key informants (three women and three men, two with extensive knowledge of conducting research within the field of FGC, all originally from different parts of Somalia and with varying years of residency in Sweden) collected data through face-to-face interviews in Somali using the questionnaire. The key informants interviewed both men and women, regardless of their own gender. The key informants were also responsible for recruiting participants, to make sure the participants accurately understood the different anatomical forms of FGC, and to strive towards establishing a trusting relationship with the participants as FGC may be a sensitive topic. Further, as it may be sensitive to disclose positive attitudes towards FGC, the key informants were individuals who are respected within the community and not associated with any authority. Prior to data collection the key informants received training by one member of the research team (AW). The training included discussions of the content of the questionnaire, and strategies for recruiting participants. Further, the training also involved discussions on the importance of having a non-judgemental position towards the attitudes expressed by the participants and to inform the participants that the information they provided would be treated with confidentiality. The key informants were also informed about the importance of conducting interviews in a private setting so that no one could overhear what was discussed.

### Definition of variables

#### Outcome variable

The outcome was measured by asking: *There are people who want female circumcision to be abolished and other people who want it to be continued. Which of the following do you want to continue?* Response alternatives were: ‘Pricking but no flesh removed,’ ‘Some flesh removed,’ ‘Flesh removed and some stitching,’ ‘Flesh removed and closed,’ and ‘All of them should be abolished’. Two participants selected several options, and their answers were recoded into the most severe type to allow data presented as one answer per participant.

#### Background variables

Background variables were: gender, age, marital status, cohabitation, education, origin, years of residency in Sweden, employment (*no work* included studying Swedish, in a programme organised by employment agency, retired/sick leave/parental leave, and unemployed), religion, own FGC/circumcision, acculturation, and measures of social capital [2, 22, 23].

Because level of acculturation after migration may affect attitudes towards FGC [24], proficiency of the Swedish language, assessed through five questions, was included as a proxy measure of *acculturation*. Response alternatives were ‘Poor,’ ‘Average,’ ‘Good,’ and ‘Very good.’ Those who answered ‘Poor’ on all five questions were categorised as having ‘Poor acculturation’; the others as having ‘Good acculturation’ [25, 26].

Social capital can be defined as ‘social networks, the reciprocities that arise from them, and the value of these for achieving (mutual) goals’. Bonding social capital is characterised by strong ties within a network that strengthen common identities. Bridging social capital is characterized by weaker ties that link people from different networks together [27]. FGC is one way to access and build social capital [23]. Moreover, what type of social capital you have may affect your attitudes towards FGC. *Social participation* was classified on the basis of participation in 13 social activities during the last year, and *trust in others* was measured through four questions yielding total scores in level of trust. Three questions measured the balance between bridging and bonding social capital in regard to trust in others, social participation, and values. These were highly correlated (Kendal’s Tau B correlation was >0.55 for all pairwise comparisons) and thus combined into *bridging social capital* [28].

#### Variables measuring attitudes and knowledge of FGC

**Fig. 1:**
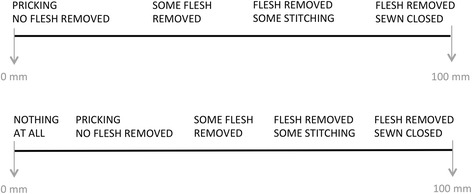
Schematic diagram over Visual Analogue Scale. Schematic diagram over Visual Analogue Scale 1 (VAS 1, *upper*) and VAS 2 (*lower*). Note: In the questionnaire only the ends of the VAS were written out and mm was not indicated

Variables assessing attitudes and knowledge of FGC were numerical and measured on two different Visual Analogue Scales (VAS) ranging from 0 to 100 millimetres (mm) to capture all different forms of FGC based on anatomy; the higher the number expressed in mm on the VAS, the more extensive form of FGC. Depending on the formulation of the question, the left end (0 mm) was marked with ‘Pricking, no flesh removed’ (VAS 1) or ‘Nothing at all’ (VAS 2) and the right end (100 mm) with ‘Flesh removed and closed’. To assist the participants to express attitudes on a VAS, a schematic diagram describing roughly the different forms of FGC based on anatomy was provided (Fig. 1). In Table 1, the numerical variables and how they were categorised are described. Further, categorical variables measured whether the participant had received information about FGC through mass media in Somalia and Sweden, and, if so, what type.

**Table 1:** Description of numerical variables measuring attitudes and knowledge of FGC (VAS measurement in mm)

Definition of FGC	VAS 1: all forms of FGC (0–10); FGC excl. pricking (11–100)
Accepted by religion	VAS 2: nothing (0); pricking (1–10); FGC excl. pricking (11–100)
Needed for respectability	VAS 2: nothing (0); pricking (1–10); FGC excl. pricking (11–100)
Violation of children’s rights	VAS 1: all forms of FGC (0–10); FGC excl. pricking (11–100); never
Long-term health complications	VAS 1: all forms of FGC (0–10); FGC excl. pricking (11–100); never
Legal in Sweden	VAS 2: nothing (0); pricking (1–10); FGC excl. pricking (11–100); don’t know

### Sample size

The outcome variable, attitudes towards continuation of FGC, provided the basis for determining sample size. The proportion expected to support the continuation of FGC was estimated at 24%, based on the first 107 collected questionnaires. The desired margin of error was chosen as 0.05, and power to 80%. To adjust for the design effect, the estimate was multiplied by 2.25, which is the average value of the design effect for the Demographic and Health Surveys indicators [29]. This gave a total sample size of 633.

### Statistical analysis

SPSS version 23 was used for all analyses. Descriptive statistics are presented as frequencies and percentages. Bivariate and multivariate logistic regression analyses were used to quantify the influence of background, attitudinal, and knowledge variables on attitudes supporting pricking. Only those who supported pricking as compared to those who thought all types of FGC should be abolished were included in the descriptive statistics and models, and participants who supported the continuation of one of the more extensive forms of FGC were excluded, as were individuals with missing data. For background variables, the Kendall’s tau-b correlation for pairwise comparisons was <0.40. Corresponding correlation for variables measuring attitudes and knowledge of FGC was <0.50, indicating a rather high pairwise correlation between some of these variables. Given these initial results, we decided to first include attitudinal and knowledge variables one-by-one to the model with the background variables (Kendall’s tau-b correlation <0.25). The criterion for statistical significance was set to 0.05. To determine which attitudinal and knowledge variable was most strongly affecting the outcome, we included all attitudinal and knowledge variables together in a forward stepwise logistic regression using *p* < 0.10 as entry and exit criteria.

## Results

### Attitudes towards continuation of FGC

Of the 648 Somali men and women living in Sweden who participated in the study, 504 (78%) reported that they wanted all forms of FGC to be abolished, 113 (18%) said they wanted pricking with no removal of flesh to continue, and 27 (4%) said they wanted one of the more extensive forms of FGC where flesh is removed with/without stiches to continue (four individuals had missing data). Note, in all subsequent descriptions and analyses, only those who supported pricking as compared to those who thought all types of FGC should be abolished were included (*n* = 617).

### Associations between background factors and support for continuation of pricking

In total, 186 (30%) lived in the municipality of Gothenburg, 188 (31%) in Malmo, 164 (27%) in Stockholm, and 79 (13%) in Uppsala. Table 2 presents background factors, portraying, in general, a heterogeneous picture. The majority were well acculturated. However, 57% had a low level of participation in social activities, 89% had a low level of trust in others, and about half had mainly within-community social capital.

**Table 2:** Background factors and odds of supporting the continuation of pricking (*n* = 617)

	Descriptive statistics^a^		Odds of supporting the continuation of pricking			
	*N* (%)	Want pricking to continue* n* (%)	cOR	95% CI	aOR^b^	95% CI
Gender						
Man	318 (51.5)	49 (15.4)	1.00	Ref.	1.00	Ref.
Woman	299 (48.5)	64 (21.4)	1.50	0.99–2.26	1.35	0.82–2.23
Age						
≤ 25	86 (14.0)	12 (14.0)	1.00	Ref.	1.00	Ref.
26–35	202 (33.0)	26 (12.9)	0.91	0.44–1.90	0.97	0.43–2.22
36–45	162 (26.4)	37 (22.8)	1.83	0.90–3.72	1.94	0.85–4.41
≥ 46	163 (26.6)	38 (23.3)	1.88	0.92–3.81	2.72*	1.14–6.48
Marital status						
Single	231 (37.6)	37 (16.0)	1.00	Ref.	1.00	Ref.
Married/Partner	330 (53.7)	64 (19.4)	1.26	0.81–1.97	1.24	0.74–2.09
Divorced/Widowed	53 (8.6)	10 (18.9)	1.22	0.56–2.64	1.24	0.51–2.99
Cohabit						
No	200 (32.6)	35 (17.5)	1.00	Ref.	–	–
Yes	413 (67.4)	76 (18.4)	1.06	0.68–1.65	–	–
Education						
University/College	59 (9.6)	7 (11.9)	1.00	Ref.	1.00	Ref.
Secondary school	201 (32.7)	29 (14.4)	1.25	0.52–3.03	1.04	0.40–2.68
Primary school	244 (39.7)	49 (20.1)	1.87	0.80–4.36	1.20	0.46–3.11
Koranic school	39 (6.4)	11 (28.2)	2.92*	1.02–8.37	1.59	0.45–5.57
No education	71 (11.6)	17 (23.9)	2.34	0.90–6.10	0.85	0.27–2.66
Somali origin						
Urban	502 (82.0)	73 (14.5)	1.00	Ref.	1.00	Ref.
Rural	110 (18.0)	39 (35.5)	3.23**	2.03–5.13	3.08**	1.81–5.25
Years of residency in Sweden						
≤ 2	155 (25.2)	45 (29.0)	3.00**	1.58–5.70	3.55**	1.52–8.29
3–4	105 (17.0)	15 (14.3)	1.22	0.57–2.63	1.51	0.60–3.78
5–9	157 (25.5)	25 (15.9)	1.39	0.70–2.76	1.82	0.82–4.06
10–14	74 (12.0)	13 (17.6)	1.56	0.70–3.50	1.72	0.71–4.17
≥ 15	125 (20.3)	15 (12.0)	1.00	Ref.	1.00	Ref.
Employment						
Work full/Part time	246 (40.1)	36 (14.6)	1.00	Ref.	1.00	Ref.
No work	328 (53.5)	70 (21.3)	1.58*	1.02–2.46	0.84	0.47–1.49
Student	39 (6.4)	6 (15.4)	1.06	0.42–2.71	1.03	0.37–2.89
Religion^c^						
Muslim	613 (99.7)	112 (18.3)	–	–	–	–
Other	2 (0.3)	0 (0.0)	–	–	–	–
FGC/circumcised^c, d^						
Yes	595 (98.3)	108 (18.2)	–	–	–	–
No	9 (1.5)	1 (11.1)	–	–	–	–
Don’t know	1 (0.2)	0 (0.0)	–	–	–	–
Acculturation						
Poor	124 (21.0)	38 (30.6)	2.35**	1.49–3.70	–	–
Good	467 (79.0)	74 (15.8)	1.00	Ref.	–	–
Social capital: Social participation						
Low	348 (56.7)	73 (21.0)	1.50	0.98–2.29	1.18	0.72–1.94
High	266 (43.3)	40 (15.0)	1.00	Ref.	1.00	Ref.
Social capital: Trust						
Low	544 (89.2)	100 (18.4)	1.01	0.52–1.97	0.82	0.40–1.70
High	66 (10.8)	12 (18.2)	1.00	Ref.	1.00	Ref.
Bridging social capital						
Non-dominant bridging	284 (46.8)	61 (21.5)	1.43	0.95–2.15	1.26	0.80–1.99
Dominant bridging	323 (53.2)	52 (16.1)	1.00	Ref.	1.00	Ref.

*CI* confidence interval, *cOR* crude odds ratio, *aOR* adjusted odds ratio, *Ref.* referent category

*p < 0.05, **p < 0.01

^a^Missing data for each variable presented ranges from 0 to 26

^b^Adjusted for gender, age, marital status, education, Somali origin, years of residency in Sweden, employment, social capital: social participation, social capital: trust, and bridging social capital. Excluded from the model were cohabit and acculturation because they highly correlated with marital status and years of residency in Sweden respectively. Valid sample size *n* = 581

^c^Excluded from the statistical models were religion and FGC/circumcised because the majority were Muslims and circumcised

^d^Includes both men and women, regardless of type of FGC/circumcision

After adjustment for all background factors, participants who were older and originated from rural areas had higher odds of supporting the continuation of pricking, compared with participants who were younger and from urban areas. Further, the odds of supporting pricking were higher among Somalis who had lived in Sweden for 2 years or less, compared with those who had resided in Sweden for 15 years or longer (Table 2).

### Associations between attitudes and knowledge of FGC and support for continuation of pricking

Descriptive statistics of attitudes and knowledge regarding FGC among Somali immigrants are presented in Table 3. About one-third did not define pricking as a form of FGC. Further, 18% said they thought pricking was acceptable to do within their religion, 11% stated that a young, unmarried woman should have at least pricking to be respectable, and 33% did not perceive pricking as a violation of children’s rights. About half stated that pricking did not cause long-term health complications. The majority (91%) knew that all forms of FGC are illegal for Swedish residents.

**Table 3:** Attitudes and knowledge regarding FGC and odds of supporting the continuation of pricking (*n* = 617)

	Descriptive statistics^a^		Odds of supporting the continuation of pricking			
	*N* (%)	Want pricking to continue* n* (%)	cOR	95% CI	aOR^b^	95% CI
Definition of FGC						
All forms of FGC	414 (67.6)	46 (11.1)	1.00	Ref.	1.00	Ref.
FGC excl. pricking	198 (32.4)	67 (33.8)	4.09**	2.68–6.26	4.18**	2.56–6.83
Accepted by religion						
Nothing	430 (70.0)	20 (4.7)	1.00	Ref.	1.00	Ref.
Pricking	108 (17.6)	39 (36.1)	11.59**	6.38–21.04	16.17**	8.02–32.61
FGC excl. pricking	76 (12.4)	53 (69.7)	47.24**	24.32–91.77	53.73**	24.96–115.68
Needed for respectability						
Nothing	386 (63.2)	22 (5.7)	1.00	Ref.	1.00	Ref.
Pricking	64 (10.5)	22 (34.4)	8.67**	4.43–16.97	10.89**	5.05–23.48
FGC excl. pricking	161 (26.4)	69 (42.9)	12.41**	7.29–21.12	15.28**	8.19–28.48
Violation of children’s rights						
All forms of FGC	378 (61.4)	24 (6.3)	1.00	Ref.	1.00	Ref.
FGC excl. pricking	206 (33.4)	88 (42.7)	11.00**	6.69–18.08	15.42**	8.27–28.77
Never	32 (5.2)	1 (3.1)	0.48	0.06–3.64	0.26	0.30–2.16
Long-term health complications						
All forms of FGC	267 (43.4)	9 (3.4)	1.00	Ref.	1.00	Ref.
FGC excl. pricking	341 (55.4)	104 (30.5)	12.58**	6.23–25.42	14.55**	6.45–32.83
Never	7 (1.1)	0 (0.0)	–	–	–	–
Legal in Sweden						
Nothing	560 (90.9)	96 (17.1)	1.00	Ref.	1.00	Ref.
Pricking	20 (3.2)	2 (10.0)	0.54	0.12–2.35	0.52	0.11–2.38
FGC excl. pricking	3 (0.5)	1 (33.3)	2.42	0.22–26.92	2.88	0.22–37.47
Don’t know	33 (5.4)	13 (39.4)	3.14**	1.51–6.53	3.01**	1.33–6.82

*CI* confidence interval, *cOR* crude odds ratio, *aOR* adjusted odds ratio, *Ref.* referent category

*p < 0.05, **p < 0.01

^a^Missing data for each variable presented ranges from 1 to 6

^b^Adjusted for gender, age, marital status, education, origin, years of residency in Sweden, employment, social capital: social participation, social capital: trust, and bridging social capital

The participants’ attitudes and knowledge of FGC were important for whether or not they supported the continuation of pricking, even after adjusting for background factors (Table 3).

To identify which attitudinal and knowledge variables were most strongly associated with the support of pricking, we used forward stepwise logistic regression. Higher odds of supporting the continuation of pricking were found among Somali immigrants who stated that they: thought pricking was acceptable to do according to their religion; did not perceive pricking as a violation of children’s rights; and did not think that pricking caused long-term health complications (Table 4).

**Table 4:** Most important motives associated with supporting the continuation of pricking (*n* = 617)

	All		Men		Women	
	aOR	95% CI	aOR	95% CI	aOR	95% CI
Accepted by religion						
Nothing	1.00	Ref.	1.00	Ref.	1.00	Ref.
Pricking	10.59**	5.44–20.62	9.18**	3.51–24.03	12.19**	4.52–32.87
FGC excl. pricking	33.48**	15.56–72.03	39.06**	12.34–123.65	38.44**	11.44–129.18
Violation of children’s rights						
All forms of FGC	1.00	Ref.	1.00	Ref.	–	–
FGC excl. pricking	2.86**	1.46–5.61	5.19**	2.09–12.87	–	–
Never	0.13	0.01–1.07	–	–	–	–
Long-term health complications						
All forms of FGC	1.00	Ref.	–	–	1.00	Ref.
FGC excl. pricking	5.52**	2.25–13.52	–	–	29.37**	3.47–248.81
Never	–	–	–	–	–	–
Definition of FGC						
All forms of FGC	–	–	1.00	Ref.	–	–
FGC excl. pricking	–	–	3.36**	1.35–8.36	–	–
Needed for respectability						
Nothing	–	–	–	–	1.00	Ref.
Pricking	–	–	–	–	4.28*	1.10–16.61
FGC excl. pricking	–	–	–	–	2.79*	1.03–7.59

*CI* confidence interval, *aOR* adjusted odds ratio, *Ref.* referent category

*p < 0.05, **p < 0.01

Included in the forward stepwise logistic regression were variables measuring attitudes and knowledge of FGC in regard to: accepted by religion, violation of children’s rights, health complications, definition, respectability, and legal in Sweden

The motives for supporting pricking differed somewhat between men and women. Religious aspects were important for both men and women. However, for men, whether they stated that pricking was a form of FGC and a violation of children’s right was associated with how they assessed the continuation of pricking. While, for women, whether they reported that pricking caused health complications and gave the woman respectability was associated with how they assessed the continuation of pricking (Table 4).

**Fig. 2:**
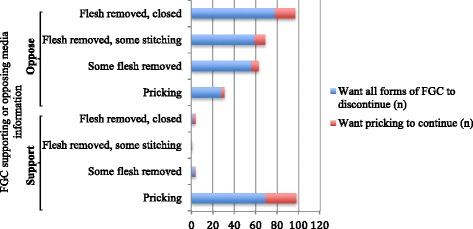
Mass media information. Type of information received through mass media in Somalia among a subset of 138 participants. Note: this was a multiple response question

In total, 54 (9%) reported that they had received information about FGC through mass media in Sweden. Among those, the majority stated that the information opposed all forms of FGC (data not shown). A larger proportion, 138 (22%), reported that they had received information about FGC through mass media in Somalia. According to them, the information, to a large extent, supported pricking, while opposing the other forms of FGC (Fig. 2).

As the data collectors were both men and women, we analysed whether the gender of the data collector may have influenced the participants’ answers. Male data collectors interviewed 180 participants; of whom 60% were men and 40% women. Female data collectors interviewed 468 participants; of whom 47% were men and 53% women. Interviews where the gender of the data collector and participant was the same had a similar proportion of participants who stated that pricking should continue as in two-gender interviews, except for a female data collector who had a higher proportion of women who said they thought pricking should continue, and a male data collector who also had a higher proportion of women who stated a support for the continuation of pricking.

Comparing the support of the continuation of pricking between the four municipalities where data were collected showed that Malmo had a significantly higher number of individuals who supported pricking compared with the other three municipalities (Pearson Chi-Square, *p* < 0.001). In a sub-analysis comparing Malmo with the other three municipalities the same trends in regard to odds of supporting pricking were noted. However, there were two exceptions where Malmo was significantly different from the other three municipalities. Women in Malmo had lower odds of supporting pricking while women in the other three municipalities had higher odds (Additional file 2), and participants in Malmo who stated that pricking was acceptable according to their religion had higher odds of supporting pricking compared with participants in the other three municipalities (Additional file 3).

## Discussion

We found that individuals assessed the practice of pricking differently despite their shared culture and religion. Somali immigrants who were older, from rural areas, and newly arrived in Sweden had higher odds of supporting pricking. Further, higher odds of supporting pricking were found among Somali immigrants who stated that they thought pricking was acceptable to do according to their religion, did not perceive pricking as a violation of children’s rights, and did not think that pricking caused long-term health complications.

Short duration of residence in a non-practising country, older age, and rural origin have been reported by others to be associated with the practice of FGC. Cultural change after migration [30], and among younger generations [24], as well as religious leaders being more likely to oppose all forms of FGC in urban areas than in rural [31], might explain this. Further, as it was generally perceived that mass media in Somalia gave a positive picture of pricking, this could have had an impact as well [32].

Religion and respectability of women were associated with the support of pricking, factors also known to be important in the assessment of other forms of FGC [33, 34]. Further, it has been suggested that having knowledge of and agreeing that FGC causes health consequences and is a violation of children’s rights is important for individuals to discontinue with FGC in general, although these aspects alone may not necessarily lead to the discontinuation of FGC [35]. Correspondingly, those in our study who stated that pricking did not have an impact on health or children’s rights had higher odds of supporting pricking. Thus, values attributed to and associated with pricking are also attributed to other, more extensive, forms of FGC. Hence, it seems that the values embedded within the practice of FGC remain somewhat unchanged, while attitudes towards what type of practice should be performed have changed. Similarly findings have also been described among Israeli Bedouins [36], where the practice of FGC changed towards less extensive or even symbolic forms, while its importance for practicing communities remained. Further, those who did not perceive pricking as a form of FGC were more likely to support pricking. This separation of pricking from FGC may be a response of moving what was a traditional practice outside the general classifications of harm. Furthermore, knowledge of health consequences of the more severe forms of FGC may have led to an increased support of pricking, a practice which many participants regarded as non-harmful.

For both Somali men and women, religion was an important aspect for supporting pricking. In Somalia, ‘Sunna’ and ‘Pharaonic circumcision’ are commonly used to define different forms of FGC. ‘Pharaonic circumcision’ usually refers to infibulation where the vaginal orifice is narrowed (FGC type III), whereas ‘Sunna circumcision’ usually refers to less extensive forms than infibulation, including or excluding pricking [37]. Thus, the use of the term ‘Sunna circumcision’ could fuel the idea that pricking is a religious Sunna (desirable to do but not a requirement). FGC is not mentioned in the Quran. However, there are a few Hadiths (recorded sayings and practices of the Prophet Mohammed) that mention the practice. One commonly cited hadith roughly translates to ‘If you cut, do not overdo it, because it [the clitoris] brings more radiance to the face, and it is more pleasant for the husband’. All of these Hadiths are judged as either unauthentic (weak) or unrelated to FGC; still, they are used by supporters of FGC to establish a link between FGC and religion [38]. This could explain why religion was strongly associated with the support of pricking in our study.

### Strengths and limitations

This study involved Somalis at several stages; in designing the study, collecting data, interpretation of the findings, and as participants. Further, the survey was conducted in four municipalities in different regions in Sweden and both men and women collected the data. This contributes to a comprehensive understanding of attitudes towards FGC among this group. From pilot studies we knew that there could be different views on what was perceived as FGC among the participants. We therefore first asked what practices they defined as FGC, and thereafter explained that subsequent questions concerning FGC referred to all forms, from pricking to infibulation. Further, survey questions were based on the anatomical extent of FGC to avoid ambiguous interpretations.

Causality was not possible to determine due to the cross-sectional study design, and there is a risk that not all confounders were accounted for. Random sampling was not used, which may affect the generalizability of the results and give a biased sample. Snowball sampling from recruited participants may lead to a more uniform sample as participants may help to recruit contacts that share the same views. As the support for the continuation of FGC among Somalis in Sweden was unknown, we based our sample size calculations on the first collected questionnaires. If this calculation instead had been based on the prevalence of FGC among Somali-born women, which is 98% [2], a larger sample size would have been required. Some odds ratios presented in the statistical models have wide confidence intervals due to few observations in some categories. For interpretation of acculturation, note that the inclusion for having poor acculturation was strict. Some participants may have been hesitant to report a supportive attitude toward FGC. Further, it could be that supportive attitudes to pricking was used as a ‘cover up’ of support of more severe forms of FGC; that it was easier to support pricking explicitly, as it has no perceived health implications, than to support one of the more severe forms. However, almost all participants knew that even pricking is illegal in Sweden; thus, if one dares to speak out about supporting pricking, one would presumably dare to say whether one also supports other forms of FGC.

## Conclusions

Pricking is an under-researched area that seems to have gained momentum among diaspora communities. We found that values known to be associated with FGC in general are also related to pricking. Hence, there seems to be a change in what types of FGC are supported rather than in their perceived value. More research, qualitative as well as quantitative, is needed to better understand the dynamics of change within a migration context and to gain a deeper understanding of attitudes towards pricking. In a globalised world, our study contributes with new knowledge on the values underpinning the practice of pricking, knowledge of importance for informed decision-making among policy-makers and health care practitioners.

## Additional files

Additional file 1: Annex 1. Questionnaire. Questionnaire used in the survey. (DOCX 106 kb)

Additional file 2: Table S1. Background factors and odds of supporting the continuation of pricking, stratified on municipality. (DOCX 21 kb)

Additional file 3: Table S2. Attitudes and knowledge regarding FGC and odds of supporting the continuation of pricking, stratified on municipality. (DOCX 71 kb)

## Abbreviations

FGC: Female genital cutting
VAS: Visual Analogue Scale
WHO: World Health Organization
