# Supplementary material for: Factors associated with the support of pricking (female genital cutting type IV) among Somali immigrants – a cross-sectional study in Sweden
Source: Reprod Health. 2017 Aug 8;14:92. doi: 10.1186/s12978-017-0351-0 (PMC5549348; doi:10.1186/s12978-017-0351-0)
Supplement: Supplementary file 1 — Questionnaire. Questionnaire used in the survey. (DOCX 106 kb) [file 12978_2017_351_MOESM1_ESM.docx]

Additional file 1

Annex 1. Questionnaire

Somali SciLive

**Baseline questionnaire**

*Chose one alternative for each question if not otherwise stated*

| IDENTIFICATION | |
| --- | --- |
| 1. | Somali organisation code ____________________ |
| 2. | Participants code ____________________ |
| 3. | Interviewers name ____________________ |
| 4. | Date (YYYY/MM/DD): ________/____/____ |
| 5. | Interview duration in minutes ____________________ |

| RESPONDENT’S BACKGROUND | | | | | |
| --- | --- | --- | --- | --- | --- |
| 6. | Sex  Man  Woman | | | | |
| 7. | What year were you born?  *As written in your passport*  Year (YYYY): ____________________ | | | | |
| 8. | How long have you lived in Sweden?  Less than 2 years  3-4 years  5-9 years  10-14 years  More than 15 years | | | | |
| 9. | **What is your level of education?**  Not been to school/ not completed any grade  Koranic school only  Primary school (1-9 years)  Secondary school (10-12 years)  College/ university | | | | |
| 10. | Before you came to Sweden, where did you grow up?  City  Village/ countryside  Nomadic life | | | | |
| 11. | What is your marital status now?  Single  Married/ in a relationship  Divorced/ widowed | | | | |
| 12. | Who lives in your home?  *Several options can be selected*  I live alone  Husband/ wife/ partner  Children  Father, mother, father in law, mother in law  Other relatives  Other, specify: __________________________________________________________________ | | | | |
| 13. | Do you have social welfare benefits (försörjningsstöd) from your municipality?  Yes  No | | | | |
| 14. | What is your main employment status?  Work full time/ part time  In a program organized by the placement service for work (Arbetsförmedlingen)  Studying Swedish (SFI, basic Swedish etc.)  Student (those who are studying something else than Swedish)  Retired/ On sick leave/ On parental leave (with parental benefits (föräldrapenning))  Unemployed | | | | |
| 15. | What is your religion?  Muslim  Christian  Atheist  Other | | | | |
| 16. | Rate your comprehension and use of the Swedish language in the following situations | | | | |
|  |  | Poor | Average | Good | Very good |
|  | (a) Ability to understand news reports on the radio and television |  |  |  |  |
|  | (b) Speaking Swedish at meetings |  |  |  |  |
|  | (c) Communicating with authorities over the telephone, (*i.e.,* calling the Health Department, Social Security Office, or Unemployment Center) |  |  |  |  |
|  | (d) Reading books in Swedish |  |  |  |  |
|  | (e) Completing a written application for employment |  |  |  |  |

| FEMALE CIRCUMCISION, BACKGROUND  Before asking the questions below, show the picture (Picture 1) of the different types of female circumcision to the respondent. Explain the different types, and that not everyone regard all these types as female circumcision. Show the other picture (Picture 2) and explain that this one also has the option Nothing at all. Have these pictures infront of the participant during the entire interview. Refer to them when there is a question starting with Picture 1 or Picture 2.  For the questions with the VAS-scale, the respondent should themselves draw the line in the questionnaire.  Make sure to explain to the respondent that the attitudes and opinions he/she express will be confidential, and that there is no right or wrong. | |
| --- | --- |
| 17. | See picture 1: What do you regard as female circumcision?  *Notera: Med denna fråga menas vilka ingrepp de tycker hör till begreppet “kvinnlig omskärelse”.*  *Note: Indicate direction*  Pricking, no Flesh removed  flesh removed and closed |
| Explain that when we use the word female circumcision in the questions below it includes all types. | |
| 18. | **Have any of your family members undergone female circumcision?**  Yes  No  Don’t know |
| 19. | Ask only women: Have you yourself been circumcised, and if so, what was done at that time?  *Note: See Picture 2.*  Yes: Pricking but no flesh removed  Yes: Some flesh removed  Yes: Flesh removed and some stitching  Yes: Flesh removed and closed  No  Don’t know  N/A |
| 20. | In Somalia, have you ever received information through mass media about female circumcision (video, TV, newspaper, internet)?  *Note: See Picture 2.*  Yes  If yes, was the information mainly:  Supporting:  Pricking but no flesh removed  Some flesh removed  Flesh removed and some stitching  Flesh removed and closed  Opposing:  Pricking but no flesh removed  Some flesh removed  Flesh removed and some stitching  Flesh removed and closed  No |
| 21. | In Sweden, have you ever received information through mass media about female circumcision (video, TV, newspaper, internet)?  *Note: See Picture 2.*  Yes  If yes, was the information mainly:  Supporting:  Pricking but no flesh removed  Some flesh removed  Flesh removed and some stitching  Flesh removed and closed  Opposing:  Pricking but no flesh removed  Some flesh removed  Flesh removed and some stitching  Flesh removed and closed  No |
| 22. | Have you yourself ever actively taken part in any group, organisation, or campaign working against female circumcision?  *Note: participation in the Somali SciLive intervention should not be registered as “Yes, in Sweden”.*  Yes, in Somalia  Yes, in Sweden  Yes, in both Somalia and Sweden  No |
| FEMALE CIRCUMCISION, DAUGHTER | |
| 23. | **See picture 2: We don’t know if you have a daughter. But let’s hypothetically say that you do have a daughter, what would you then do?**  Nothing at all Flesh removed  and closed |
| 24. | How many within the Somali community in Sweden do you think circumcise their daughters?  No one Everybody |
| FEMALE CIRCUMCISION, ATTITUDES | |
| 25. | **See picture 2: What do you think is acceptable to do?**  *Notera: Här ska den intervjuade svara på vilka typer av ingrepp han/hon tycker är acceptabla att utföra.*  *Note: Indicate direction*  Nothing at all Flesh removed  and closed |
| 26. | **See picture 2: What do you think most Somali men in Sweden think is acceptable to do?**  *Note: Indicate direction*  Nothing at all Flesh removed  and closed |
| 27. | **See picture 2: What do you think most Somali women in Sweden think is acceptable to do?**  *Note: Indicate direction*  Nothing at all Flesh removed  and closed |
| 28. | See picture 2: What do you think is accepted to do according to your religion?  *Note: Indicate direction*  Nothing at all Flesh removed  and closed |
| 29. | See picture 1: When, if ever, will the circumcision cause long-term health complications for girls/women?  *Note: Indicate direction*  Never  Pricking, no Flesh removed  flesh removed and closed |
| 30. | See picture 1: When, if ever, will the circumcision become a violation of children’s rights?  *Note: Indicate direction*  Never  Pricking, no Flesh removed  flesh removed and closed |
| 31. | See picture 2: To be a seen as a respectful not yet married young woman, what is needed according to you?  *Note: Indicate direction*  Nothing at all Flesh removed  and closed |
| 32. | **What benefits do girls themselves get if they undergo circumcision?**  *Several options can be selected*  Cleanliness/hygiene  Social acceptance  Better marriage prospects  Preserve virginity/prevent premarital sex  More sexual pleasure for man  Religious approval  Other, specify:______________________________________________________________________  No benefits |
| 33. | **What benefits do girls themselves get if they do NOT undergo circumcision?**  *Several options can be selected*  Fewer medical problems  Avoiding pain  More sexual pleasure for her  More sexual pleasure for the man  Follows religion  Other, specify:_____________________________________________________________________  No benefits |
| 34. | **There are people who want female circumcision to be abolished and other people who want it to be continued. What of the following do you want to continue?**  *Note: Several options can be selected. See Picture 1.*  Pricking but no flesh removed  Some flesh removed  Flesh removed and some stitching  Flesh removed and closed  All of them should be abolished |
| 35. | Do you think it is a good idea for men to be involved in the debate on female circumcision?  Yes  No |
| 36. | Ask only men: For your marriage, do you prefer a woman who is circumcised or one who is not circumcised?  *Note: See Picture 2.*  Not circumcised  Circumcised: Pricking but no flesh removed  Circumcised: Some flesh removed  Circumcised: Flesh removed and some stitching  Circumcised: Flesh removed and closed  It doesn’t matter  N/A |
| 37. | Ask only women: Do you think Somali men prefer to marry a woman who is circumcised or one who is not circumcised?  *Note: See Picture 2.*  Not circumcised  Circumcised: Pricking but no flesh removed  Circumcised: Some flesh removed  Circumcised: Flesh removed and some stitching  Circumcised: Flesh removed and closed  It doesn’t matter for them  N/A |
| FEMALE CIRCUMCISION, KNOWLEDGE | |
| 38. | **See picture 2: What is legal to do in Sweden?**  *Note: Indicate direction*  Don’t know  Nothing at all Flesh removed  and closed |
| 39. | Living in Sweden, is it legal to take a girl abroad for circumcision?  Yes  No  Don’t know |

| MALE CIRCUMCISION | |
| --- | --- |
| 40. | Ask only men: Have you yourself been circumcised?  Yes  No  Don’t know  N/A |
| 41. | Do you see any reason for questioning circumcision of boys?  *Notera: Här ska den intervjuade svara på om han/hon ser några skäl till att inte utföra manlig omskärelse, om han/hon ser något skäl till att ifrågasätta utförandet av manlig omskärelse.*  Yes  No |
| 42. | Some people say that male circumcision is a violation of children’s rights, do you agree?  Yes  No |
| 43. | In regard to physical health, where would you place the effects of male circumcision?  *Notera: Om sträcket dras precis i mitten innebär det att den intervjuade varken ser några fördelaktiga eller skadliga effekter av manlig omskärelse. Om strecket dras mer till vänster innebär det att den intervjuade tycker att det finns fler fördelaktiga effekter, och om strecket dras mer till höger så tycker den intervjuade att det finns fler skadliga effekter.*  Only Only  beneficial harmful |
| 44. | In your opinion, are circumcision of girls and boys comparable practices?  *Note: Several options can be selected. See Picture 2.*  Yes, pricking but no flesh removed is comparable with male circumcision  Yes, some flesh removed is comparable with male circumcision  Yes, flesh removed and some stitching is comparable with male circumcision  Yes, flesh removed and closed is comparable with male circumcision  No, none of them are comparable with male circumcision |

| SOCIAL CAPITAL  Before asking the questions below, explain for the respondent that you now will ask some questions about his/her daily life in Sweden. | | | | | |
| --- | --- | --- | --- | --- | --- |
| 45. | Make a decision about the following statements | | | | |
|  |  | I do not agree at all | I do not agree | I agree | I agree completely |
|  | (a) Most people would take advantage of you if they had an opportunity |  |  |  |  |
|  | (b) Most people try to be fair |  |  |  |  |
|  | (c) You can trust most people |  |  |  |  |
|  | (d) You cannot be careful enough when dealing with other people |  |  |  |  |
| **46.** | Have you during the last 12 months…  *Note: The following applies only during the time spent in Sweden. Several options can be selected.*  Participated in a study circle/course  Participated in a union meeting  Participated in a meeting of Somali organizations  Participated in a meeting of other organizations  Been to the theatre/cinema  Been at a cultural event/activity  Participated in a religious event  Been at a sports event  Participated in a celebration of the Somali independence day  Participated in a demonstration of any kind  Visited a public event, e.g. music concert, entertainment or similar  Participated in a big gathering of relatives  Been at a private party or wedding/engagement party  None of the above | | | | |
| **47.** | **During your social activities, how many of the other participants in those activities are of the same background (e.g. sex, education or country of origin) as your own?**  *Note: Chose one alternative*  All the other participants in those activities have the same background as my own  Most of the other participants in those activities have the same background as my own  About half of the participants in those activities have the same background as my own  Most of the other participants in those activities have a different background than my own  All the other participants in those activities have a different background than my own | | | | |
| **48.** | Does a person’s background (e.g. sex, education, or country of origin) affect your level of trust for them, for example the credibility for what they say about different things?  *Note: Chose one alternative*  I only trust persons with the same background as my own  I trust persons with the same background as my own rather more than others  I trust persons with the same background as my own a bit more than others  I trust persons with the same background as myself equally as much as others  I trust persons with the same background as myself less than others | | | | |
| **49.** | **How well do your common opinions and values match other persons of the same background (e.g. sex, education or country of origin) as your self?**  *Note: Chose one alternative*  All my opinions and values are the same as other persons’ with the same background as my own  Most of my opinions and values are the same as other persons’ with the same background as my own  About half of my opinions and values are about the same as other persons' with the same background as my own  Most of my opinions and values differ compared with other persons’ with the same background as my own | | | | |
