# Supplementary material for: Factors associated with the support of pricking (female genital cutting type IV) among Somali immigrants – a cross-sectional study in Sweden
Source: Reprod Health. 2017 Aug 8;14:92. doi: 10.1186/s12978-017-0351-0 (PMC5549348; doi:10.1186/s12978-017-0351-0)
Supplement: Supplementary file 2 — Background factors and odds of supporting the continuation of pricking, stratified on municipality. (DOCX 21 kb) [file 12978_2017_351_MOESM2_ESM.docx]

Additional file 2

| Table 5. Background factors and odds of supporting the continuation of pricking, stratified on municipality | | | | | |
| --- | --- | --- | --- | --- | --- |
|  | Malmo | |  | Gothenburg, Stockholm, Uppsala | |
|  | aOR^1^ | 95% CI |  | aOR^1^ | 95% CI |
| Gender |  |  |  |  |  |
| Man | 1.00 | Ref. |  | 1.00 | Ref. |
| Woman | 0.40* | 0.17–0.94 |  | 2.41* | 1.14–5.08 |
| Age |  |  |  |  |  |
| ≤ 25 | 1.00 | Ref. |  | 1.00 | Ref. |
| 26­–35 | 0.35 | 0.10–1.22 |  | 1.61 | 0.37–7.09 |
| 36–45 | 0.76 | 0.21–2.77 |  | 4.63* | 1.11–19.34 |
| ≥ 46 | 1.03 | 0.26–4.10 |  | 7.26** | 1.61–32.78 |
| Marital status |  |  |  |  |  |
| Single | 1.00 | Ref. |  | 1.00 | Ref. |
| Married/Partner | 1.65 | 0.71–3.86 |  | 0.80 | 0.37–1.73 |
| Divorced/Widowed | 0.78 | 0.18–3.33 |  | 1.40 | 0.41–4.79 |
| Education |  |  |  |  |  |
| University/College | 1.00 | Ref. |  | 1.00 | Ref. |
| Secondary school | 1.78 | 0.46–6.95 |  | 0.93 | 0.22–3.95 |
| Primary school | 3.11 | 0.77–12.52 |  | 1.12 | 0.25–4.97 |
| Koranic school | 5.65 | 0.51–62.68 |  | 2.27 | 0.38­–13.46 |
| No education | 1.47 | 0.26–8.34 |  | 0.98 | 0.18–5.42 |
| Somali origin |  |  |  |  |  |
| Urban | 1.00 | Ref. |  | 1.00 | Ref. |
| Rural | 1.87 | 0.81–4.30 |  | 4.29* | 1.80–10.22 |
| Years of residency in Sweden |  |  |  |  |  |
| ≤ 2 | 1.76 | 0.45–6.89 |  | 2.85 | 0.70–11.52 |
| 3–4 | 1.40 | 0.31–6.37 |  | 1.42 | 0.35–5.69 |
| 5–9 | 1.40 | 0.37–5.30 |  | 2.64 | 0.84–8.31 |
| 10–14 | 1.21 | 0.23–6.32 |  | 2.77 | 0.86–8.95 |
| ≥ 15 | 1.00 | Ref. |  | 1.00 | Ref. |
| Employment |  |  |  |  |  |
| Work full/Part time | 1.00 | Ref. |  | 1.00 | Ref. |
| No work | 1.42 | 0.48–4.23 |  | 0.48 | 0.21–1.13 |
| Student | 0.65 | 0.16–2.69 |  | 1.18 | 0.20–6.98 |
| Social capital: Social participation |  |  |  |  |  |
| Low | 1.65 | 0.68–3.98 |  | 1.23 | 0.59–2.55 |
| High | 1.00 | Ref. |  | 1.00 | Ref. |
| Social capital: Trust |  |  |  |  |  |
| Low | 1.04 | 0.34–3.21 |  | 0.83 | 0.26–2.68 |
| High | 1.00 | Ref. |  | 1.00 | Ref. |
| Bridging social capital |  |  |  |  |  |
| Non-dominant bridging | 1.81 | 0.86–3.83 |  | 1.29 | 0.62–2.68 |
| Dominant bridging | 1.00 | Ref. |  | 1.00 | Ref. |

CI, confidence interval; aOR, adjusted odds ratio, Ref., referent category

* *p* < 0.05, ** *p* < 0.01

^1^ Adjusted for gender, age, marital status, education, Somali origin, years of residency in Sweden, employment, social capital: social participation, social capital: trust, and bridging social capital
