# Supplementary material for: Factors associated with the support of pricking (female genital cutting type IV) among Somali immigrants – a cross-sectional study in Sweden
Source: Reprod Health. 2017 Aug 8;14:92. doi: 10.1186/s12978-017-0351-0 (PMC5549348; doi:10.1186/s12978-017-0351-0)
Supplement: Supplementary file 3 — Attitudes and knowledge regarding FGC and odds of supporting the continuation of pricking, stratified on municipality. (DOCX 71 kb) [file 12978_2017_351_MOESM3_ESM.docx]

Additional file 3

| Table 6. Attitudes and knowledge regarding FGC and odds of supporting the continuation of pricking, stratified on municipality | | | | | |
| --- | --- | --- | --- | --- | --- |
|  | Malmo | |  | Gothenburg, Stockholm, Uppsala | |
|  | aOR^1^ | 95% CI |  | aOR^1^ | 95% CI |
| Definition of FGC |  |  |  |  |  |
| All forms of FGC | 1.00 | Ref. |  | 1.00 | Ref. |
| FGC excl. pricking | 1.60 | 0.74–3.45 |  | 4.73** | 2.16–10.36 |
| Accepted by religion |  |  |  |  |  |
| Nothing | 1.00 | Ref. |  | 1.00 | Ref. |
| Pricking | 88.82** | 21.63–364.64 |  | 6.64** | 2.26–19.47 |
| FGC excl. pricking | 52.70** | 13.22–210.06 |  | 55.68** | 18.69–165.90 |
| Needed for respectability |  |  |  |  |  |
| Nothing | 1.00 | Ref. |  | 1.00 | Ref. |
| Pricking | 6.57** | 1.98–21.87 |  | 15.02** | 4.82–46.77 |
| FGC excl. pricking | 10.20** | 3.97–26.25 |  | 19.69** | 7.44–52.12 |
| Violation of children’s rights |  |  |  |  |  |
| All forms of FGC | 1.00 | Ref. |  | 1.00 | Ref. |
| FGC excl. pricking | 15.66** | 5.74–42.77 |  | 13.54** | 5.55–33.01 |
| Never | – | – |  | 0.37 | 0.04–3.64 |
| Long-term health complications |  |  |  |  |  |
| All forms of FGC | 1.00 | Ref. |  | 1.00 | Ref. |
| FGC excl. pricking | 4.04* | 1.03–15.92 |  | 24.99** | 7.91–78.94 |
| Never | – | – |  | – | – |
| Legal in Sweden |  |  |  |  |  |
| Nothing | 1.00 | Ref. |  | 1.00 | Ref. |
| Pricking | 2.48 | 0.08–76.95 |  | 0.56 | 0.06–5.39 |
| FGC excl. pricking | – | – |  | 45.87* | 2.05–1024.71 |
| Don’t know | – | – |  | 14.92** | 5.08–43.81 |

CI, confidence interval; aOR, adjusted odds ratio; Ref., Referent category

* *p* < 0.05, ** *p* < 0.01

^1^ Adjusted for gender, age, marital status, education, origin, years of residency in Sweden, employment, social capital: social participation, social capital: trust, and bridging social capital
